# Nutritional knowledge and practices of low-income women during pregnancy: a qualitative study in two Oaxacan cities

**DOI:** 10.1186/s41043-025-00776-8

**Published:** 2025-02-07

**Authors:** Marian Marian, Ramona L. Pérez, Amanda C. McClain, Samantha Hurst, Elizabeth Reed, Kathryn M. Barker, Rebecka Lundgren

**Affiliations:** 1 Herbert Wertheim School of Public Health and Human Longevity Science, University of California, San Diego, CA USA; 2 San Diego State University School of Public Health, San Diego, CA USA; 3 Department of Anthropology, San Diego State University, San Diego, CA USA; 4 School of Exercise and Nutritional Sciences, San Diego State University, San Diego, CA USA; 5 Center on Gender Equity and Health, University of California, San Diego, La Jolla, CA USA

**Keywords:** Mexico, Nutrition, Nutritional education, Prenatal care, Nutritional supplements, Pregnancy, Nutritional knowledge, Nutrition practices

## Abstract

**Background:**

Adequate dietary intake is critical for healthy pregnancies. Recent changes in social services in Mexico, coupled with high levels of food insecurity, call into question whether expecting women of the lowest socioeconomic status are able to meet their dietary and nutritional needs in this changing context. The aim of this study was to explore the nutritional practices, education and received and employed among women during their pregnancy.

**Methods:**

Guided by Ecological Systems Theory and an Intersectionality Framework, this qualitative study was carried out in Oaxaca City and Puerto Escondido, in Oaxaca, a Mexican state with high levels of food insecurity. Women who had at least one child in the past five years and had lived in Oaxaca for the past five years were eligible to participate. Twenty-five women participated in semi-structured in-depth interviews conducted between June and December of 2023. A grounded theory approach was used for coding. NVivo was used for coding and analyses.

**Results:**

Five key themes emerged linked to individual-level characteristics and the multiple social identities related to the social support for nutritional knowledge and practices among low-income Oaxacan women during pregnancy: (1) Life experiences, sociodemographic, and health characteristics that influence nutritional practices and knowledge during pregnancy; (2) Female family members as a primary source of nutritional knowledge and food support; (3) Support from other members of women’s social networks; (4) Medical guidance for nutrition during pregnancy; and (5) Quality and gaps in the broader health care system and social services. These themes highlight how women’s own experiences and social identities and the different interpersonal and community-level environments, particularly those of mothers and grandmothers and health care providers, interact and shape women’s nutritional knowledge and practices, such as foods and nutritional supplements consumed, during pregnancy.

**Conclusion:**

Nutritional knowledge and practices during pregnancy are impacted by multiple social identities women have and different factors at the individual, group, and structural level. Future multi-level approaches in research and programming that take into consideration the individual, family members, and other social influences are needed to address the gaps in nutrition that women in Oaxaca go through during the prenatal period.

**Supplementary Information:**

The online version contains supplementary material available at 10.1186/s41043-025-00776-8.

## Introduction

Healthy maternal nutrition is vital for fetal development along with short- and long-term intergenerational health outcomes for the mother and the child [1–4]. Adequate nutrition can be challenging for women who do not have proper access to affordable food as they struggle to meet the increased nutrient and energy needs during the antenatal period [5, 6]. Food insecurity during pregnancy affects several health outcomes for the mother and the developing child, including increasing the risk of low birth weight and certain birth defects in children and a greater risk of chronic diseases later in life for these children [4, 7, 8]. Food insecurity in the form of reduced variety or quality of food among expecting mothers is associated with poor physical and mental health and a reduced quality of life for women [4]. Other examples of health conditions in women that experience food insecurity during pregnancy include depression, anxiety, higher gestational weight gain, severe pregravid obesity, disordered eating, gestational diabetes mellitus, and higher dietary fat intake [4, 9, 10]. Food insecurity is also associated with pregnancy-related mortalities [4]. A healthy diet during pregnancy, along with the recommended daily dose of nutritional supplements, specifically iron and folic acid, is essential in decreasing the risk to maternal health and infant low birth weight, along with other short- and long-term health issues for mothers and newborns [11, 12]. 

Food insecurity is pervasive in Mexico, as almost 34% of the population experiences some form of food insecurity, and 6.4% are extremely food insecure [13]. Between the years 2018 and 2020, food insecurity in Mexico increased by more than 5% [14], with women of reproductive age being one of the most affected by food insecurity in this country [15]. The dietary transition in Mexico in the past few decades has resulted in people eating more foods of low nutritional value, such as highly processed foods, sugars, and saturated fats, rather than the historic *dieta de la milpa*, translated as a diet based on the triad of corn, beans, and squash along with chilies, fruit, and dark leafy greens [16]. These dietary shifts do not sufficiently meet the daily nutritional needs required during pregnancy [17–19]. According to recent research, the Mexican population consumes 25% of the recommended amount of legumes and 50% of the recommended amount of fruit and vegetables [20]. The dietary consequences of food insecurity for pregnant women are typically illustrated by the prevalence of micronutrient deficiencies, primarily iron [21]. The national prevalence of anemia, a health condition that develops due to insufficient iron, among pregnant women has been documented at 35%, compared to almost 18% among non-pregnant women [22]. At the state level, the prevalence of anemia goes as high as almost 48% [23]. The World Health Organization (WHO) strongly recommends pregnant women take daily oral iron and folic acid supplements as part of their antenatal care to reduce the risk of low birth weight, maternal anemia, and iron deficiency [24]. In the early 2010s, 97% of women who received prenatal care in Mexico were prescribed folic acid, the highest among all services provided during prenatal care in the country, while 94% of women were prescribed iron, vitamins, or other nutritional supplements as well [25]. Up to 2019–2020, national social programs such as *Prospera* (formerly *Oportunidades* (2002–2014) and *Progresa* (1997–2002)) provided nutritional supplements to pregnant and lactating women living in extreme poverty [26]. While *Seguro Popular*, Mexico’s former public health insurance program for people without private or public social security system health insurance, through its *Embarazo Saludable* (Healthy Pregnancy) strategy, provided free-of-charge healthcare for women through the prenatal and delivery period [27, 28]. Previous literature has found that both *Prospera* and *Seguro Popular* had a positive impact on nutritional and natal delivery indicators of both women and infants [29, 30]. However, for the past five years, the country’s political transition has reshaped the social program structure in Mexico and eliminated both *Prospera* (2014–2019) and *Seguro Popular* (2003–2020) [26, 27]. 

The southwestern state of Oaxaca in Mexico is one of the states with a higher level of food insecurity and poverty in the country. Oaxaca is the third most economically marginalized state among the 32 federative entities that make up the country, with more people living in poverty (58%), as determined by the national poverty line [31, 32]. Additionally, 20% of the people in Oaxaca live in extreme poverty, compared to 7% of the national population [13, 33, 34]. Oaxaca has the second-highest percentage of gaps in healthcare access and education in the country [34]. Almost 85% of the population in Oaxaca does not have access to health services, and more than 60% of the population in Oaxaca does not have access to basic housing services [35]. Oaxaca also had the lowest score of the country related to the 2021 social progress index [34]. Specific to food and nutrition, as of 2018, 57.4% of the population in Oaxaca experienced some form of food insecurity, with 27.9% experiencing moderate or severe food insecurity [36]. While lack of food access, which corresponds to constant moderate or severe persistent restrictions of sufficient food intake for an active and healthy life, in Oaxaca decreased by 0.7% between 2008 and 2018, this is a lower improvement rate compared to the national level for that same period of time (1.3%), and the lack of food access, defined as the population with moderate or severe food insecurity, is 7.5% higher in Oaxaca than at the national level [36]. The number of people who cannot afford the national basic food basket in Oaxaca increased by 4.5% between 2008 and 2018, with a slight improvement from 2016 to 2018 [36]. 

To our knowledge, there are only a limited number of publications in the literature that have explored the socio-cultural contexts of pregnancy among Mexican women [37]. Limited research exists on the nutritional knowledge and practices of pregnant women in Mexico, with existing literature mostly focusing on macronutrient intake and breastfeeding [38–43]. While Mexico’s 2022 National Health and Nutrition Survey states that 83% of interviewed women received healthy eating advice from medical professionals during the pregnancy and the breastfeeding period [44], little is known about all of the sources from where women acquire the prenatal nutritional knowledge and the actual nutritional practices of women during pregnancy. This results in a critical gap in the evidence base, limiting the understanding of the barriers that pregnant women may experience while trying to meet their dietary needs, as well as the nutritional resources and facilitators these women rely on during this important and critical period for the mother and child. In response to these gaps, we employed a qualitative research design to interview low-income women with a recent pregnancy (≤ 5 years) within two cities in the state of Oaxaca. The study aimed to identify and describe the nutrition behaviors and experiences during pregnancy among low-income women in two cities of the state of Oaxaca. The research focused on the prenatal supplemental acquisition and use behaviors during the prenatal period, nutritional practices during pregnancy, and the nutritional education and knowledge acquired during pregnancy, as well as understanding the similarities and differences in these nutritional knowledge and practices during pregnancy among low-income women residing in two different urban regions in Oaxaca.

## Methods

### Conceptual framework

**Fig. 1:**
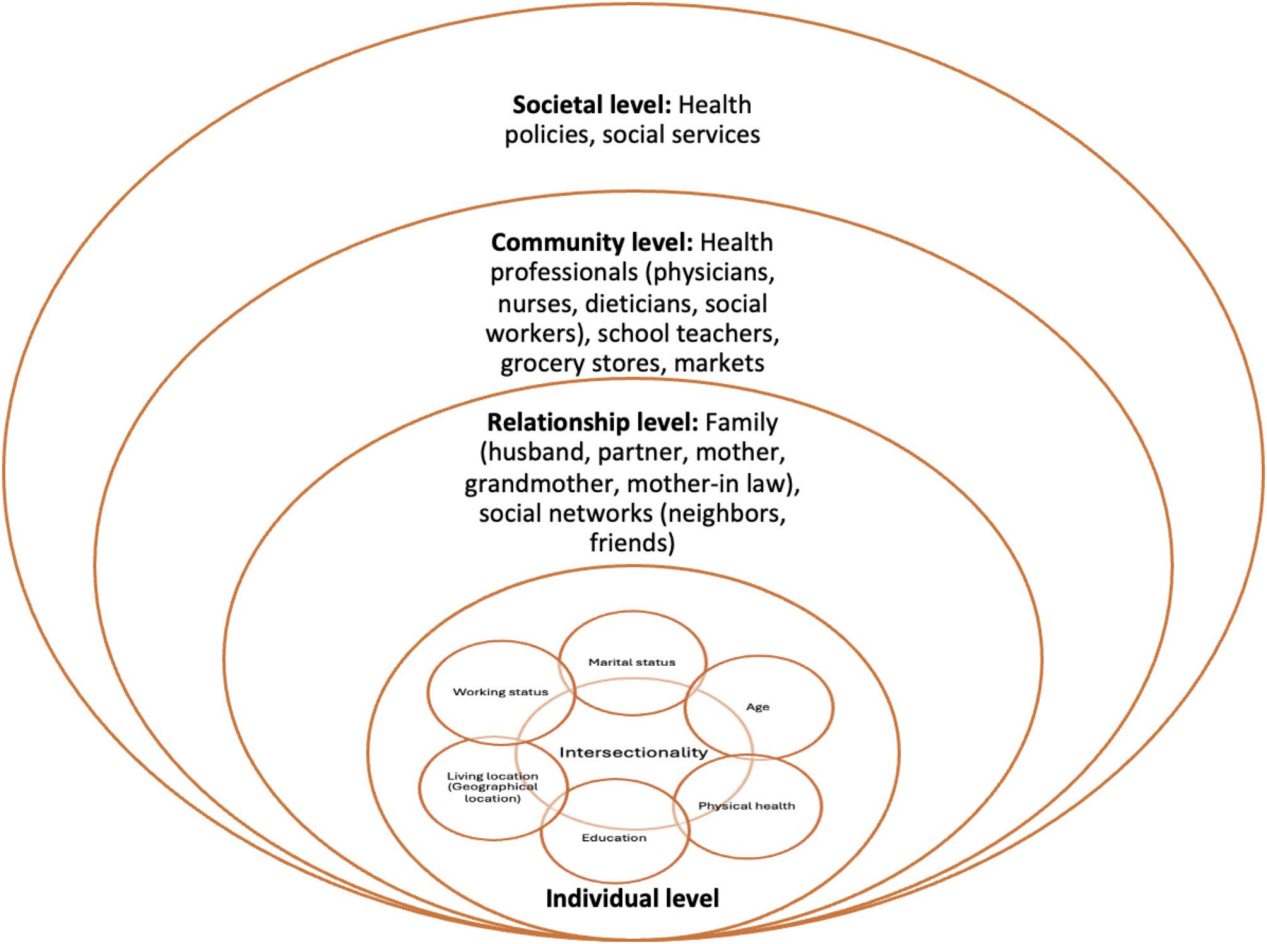
Adaptation from the socio-ecological model and intersectionality framework depicting the nutritional knowledge and practices among Oaxacan women during pregnancy

The Ecological Systems Theory [45] and the Intersectionality Framework [46] were used for this research to better understand how different relationship levels influence the nutritional knowledge and practices of the participants during pregnancy and to analyze the complexities that these women, as a result of their multiple social positions, experience in health, specifically related to nutrition during pregnancy. Using both frameworks together provides a more nuanced understanding of the intersection and interaction between individual-level characteristics and the multiple social identities in the nutritional knowledge and practices of pregnant Oaxacan women. Figure 1 illustrates an adaptation of the socio-ecological model and the intersectionality framework [47–49] depicting factors influencing nutritional knowledge and practices among Oaxacan women during pregnancy.

### Study settings

This study was conducted in the cities of Oaxaca and Puerto Escondido, in the state of Oaxaca. Two different urban cities were chosen as the setting of this study in order to capture the population diversity at the urban level in this southwestern state. Geographically, the state of Oaxaca is divided into seven regions. The city of Oaxaca, which is also the state capital, is located in Valles Centrales, while Puerto Escondido is in the Costa region. Oaxaca City and Puerto Escondido have the first and third highest population concentrations in the state, respectively [50, 51]. The population in Oaxaca City is close to 271,000, with 8% of them speaking an Indigenous language [52], while 42,000 live and 3% speak an Indigenous language in Puerto Escondido [53, 54]. The main economic source for both cities is tourism [55]. Oaxaca’s cultural diversity, the different Indigenous cultures, and its post-colonial history have guided the local and regional state’s culinary practices [56–59]. The primary diet of people in the state of Oaxaca is based on maize, beans, and peppers [57, 58]. Specifically in the city of Oaxaca, vegetables are a main staple in their diet due to the high variety of these foods in the region [57, 58]. While in Puerto Escondido, seafood, mainly fish and shellfish, is the main part of the diet of the people due to the geographic location next to the sea [60]. The food costs and practices have also been impacted in both cities, as foreign immigration has increased considerably in the last twenty years, particularly in Oaxaca City [61, 62]. For example, a recent study found that the most consumed foods during pregnancy for women who received their prenatal care at health centers in Puerto Escondido include tortillas (84%), maize-derived products (85%), and dairy products (88%) [63]. However, compared with pregnant women from rural Oaxaca, women in Puerto Escondido consumed more meats, pizza, soda, and tortas (meal similar to a sandwich) during their prenatal period [63]. Still, the state of Oaxaca, along with the rest of the country, is facing a nutritional transition that has increased malnutrition and metabolic diseases in the urban and rural Oaxacan population [64]. While the typical diet in the state of Oaxaca, the *dieta de la milpa*, is nutritious and based on the regional characteristics and the culture of this region [65], the nutritional transition that Oaxaca is facing and high levels of food insecurity in the state [36, 66] present a complex landscape to understand.

### Participant recruitment

Women who were at least 18 years of age at the time of the interview, had at least one full-term pregnancy in the last five years, and had lived in the state of Oaxaca full-time during the last five years were eligible to participate in this study. Different sampling strategies were used between the two sites based on the access to eligible women for recruitment suggested or provided by local stakeholders in each city. A combination of opportunistic and snowball sampling —the identification and recruitment of new participants by other participants —was used in the city of Oaxaca to recruit participants for these interviews. The primary recruitment techniques used were study flyers posted at local health centers and hospitals, in-person solicitation for potential participants at markets, hospitals, and health centers, phone calls to health center’s patients, and word of mouth among participants. For most of the recruitment process in the city of Oaxaca, a brief telephone screening was completed to determine eligibility. An in-person meeting was then scheduled via phone call, text message, or WhatsApp message. In Puerto Escondido, opportunistic sampling of women visiting a health center was used exclusively for recruitment. Due to participants’ convenience, some of the recruitment in the city of Oaxaca and all recruitment in Puerto Escondido consisted of back-to-back screening, consenting, and completion of the interview at the location of recruitment.

### Ethical consideration

This study was approved by the San Diego State University Institutional Review Board (Record Number HS-2023-0108). Written informed consent was obtained by all participants after a thorough explanation of the purpose of the study.

### Data collection

A semi-structured interview guide with open-ended questions and probes was used to explore the topics of focus on diet changes during pregnancy, nutrition education during pregnancy, food acquisition during pregnancy, consumption of nutritional supplements during pregnancy, and access and use of social services during pregnancy (See Supporting material 1). The development of the semi-structured interview guide was guided by the Ecological Systems Theory and the Intersectionality Framework [45, 46] along with previous work with these populations by the primary investigators (MM and RLP).

The semi-structured interviews were completed between June and December 2023. The majority of interviews in the city of Oaxaca (*n* = 6) were completed at the San Diego State University Oaxaca Center for Mesoamerican Studies, while the rest of interviews (*n* = 3) in this city were completed at the place where participants were recruited (e.g., markets, local events). The interviews in the city of Puerto Escondido (*n* = 16) were completed at the Lázaro Cárdenas Health Center.

Before the interview, participants were asked to complete the 8-item adult version of the Latin American and Caribbean Scale of Food Security (ELCSA, is the Spanish acronym) [67] to determine their food security status and a short socio-demographic survey that collected information on age, marital status, highest educational attainment, employment status, Indigenous belonging, language spoken at home, place of birth, number of years living in Oaxaca City/Puerto Escondido, type of health insurance, number of pregnancies, and the years when they gave birth. All semi-structured in-depth interviews were conducted by the primary investigator (MM), a native Spanish speaker with experience in qualitative data collection methods. Interviews were conducted in Spanish, the primary language of all the participants, and audio-recorded in the original language for transcription. Interviews lasted between 30 and 45 min and involved ice-breaker questions, followed by the interview questions from the guide, and finishing with member-checking and discussing any question or additional information the participant wanted to mention. Field notes were created by the interviewer after each interview was completed, and the notes were debriefed with another trained researcher (RLP). No new themes emerged after completing 25 in-depth interviews, suggesting saturation was reached [68]. 

### Data analysis

Interviews were transcribed verbatim (by the primary author, Microsoft teams, or a professional transcription services company), coded, and analyzed in Spanish to maintain the richness of the language and contribute to the trustworthiness of qualitative research [69]. Identifiers were removed by the primary author before analyses. Data collection and data analysis were completed concurrently by the primary investigator (MM) to tailor the interview guide, develop the codebook, and identify emergent themes. Data coding and analysis were completed independently by the primary investigator and a graduate student researcher (LC), both fluent in Spanish and with experience in qualitative methods and nutrition research in the state of Oaxaca. Data analysis was also supported by two faculty members (RLP and SM), experts in qualitative methods. A grounded-theory approach [70, 71] was used for the analysis through emic coding, as the codes used were derived from the data. The Ecological Systems Theory and the Intersectionality Framework were also incorporated into the development of the codebook and dissemination of results, as these methodological frameworks guided the entire research process. An initial codebook was created by the primary investigator after reviewing the data to identify emerging codes. The codebook evolved through the coding processes of the two coders, as new codes were added with the consensus of the coders; however, no new codes emerged after half of the transcriptions were coded, suggesting saturation was reached. Frequent discussions between the two coders during the coding process were done in order to achieve inter-rater reliability. NVivo R1 was the software used for the coding and analysis of transcripts.

## Results

### Participant characteristics

Twenty-five mothers completed semi-structured in-depth interviews. As shown in Table 1 below, the majority of women were either married or living with a partner (*n* = 22). Most women had an educational attainment of middle school or lower (*n* = 19), were stay-at-home mothers (*n* = 17), and reported having had between 1 and 3 pregnancies (*n* = 23). Almost all participants were born in the state of Oaxaca (*n* = 23) and the majority were food insecure (*n* = 20).

**Table 1:** Characteristics of women participating in semi-structured interviews in two cities in Oaxaca (*n* = 25)

Characteristic	*n* (%)	Characteristic	*n* (%)
Age, y		**Place of birth**	
20–25	8 (32%)	State of Oaxaca	23 (92%)
26–30	5 (20%)	Mexico City	1 (4%)
31–35	6 (24%)	Baja California	1 (4%)
36–40	3 (12%)	**Current health insurance**	
40–42	3 (12%)	No	23 (92%)
Marital Status		Yes	2 (8%)
Lives with partner	12 (48%)	**Number of pregnancies**	
Married	10 (40%)	1	7 (28%)
Single	3 (12%)	2	9 (36%)
Highest Educational attainment		3	7 (28%)
No education	1 (4%)	5–7	2 (8%)
Elementary school	4 (16%)	**Food security status** ^*^	
Middle school	14 (56%)	Food secure	5 (20%)
High school	5 (20%)	Low food insecurity	15 (60%)
University degree	1 (4%)	Moderate food insecurity	4 (16%)
Current employment		Severe food insecurity	1 (4%)
Stay at home	17 (68%)	**Indigenous belonging**	
Employed	8 (32%)	Yes	14 (56%)
		No	11 (44%)

^*^Measured using the eight-item adult version of the Latin American and Caribbean Food Security Scale (ELCSA) survey

### Qualitative findings

Driven by the Ecological Systems Theory and the Intersectionality Framework, we identified five themes linked to individual-level characteristics and the multiple social identities related to the social support for nutritional knowledge and practices among low-income Oaxacan women during pregnancy. The five themes that emerged were: (1) Life experiences, sociodemographic, and health characteristics that influence nutritional practices and knowledge during pregnancy; (2) Female family members as a primary source of nutritional knowledge and food support; (3) Support from husbands and other members of women’s social network; (4) Medical guidance for nutrition during pregnancy; and (5) Quality and gaps in the broader health care system and social services. Each theme, along with supportive quotes, is presented next. See Supporting Material 2 for a table of additional quotes representative of each of the themes.

#### Theme 1. Life experiences, sociodemographic, and health characteristics that influence nutritional practices and knowledge during pregnancy

The first theme relates to what women ate during their pregnancy, how they obtained what they ate, their use of nutritional supplements, nutritional knowledge gained through education and life experience, and different decisions and experiences that shaped these practices during the prenatal period.

Related to nutrition practices, the food eaten during pregnancy among women in the cities of Oaxaca and Puerto Escondido were similar. Fruits, vegetables, beans, and lentils were the most common foods mentioned by participants that were consumed during pregnancy. Examples of vegetables included potatoes, broccoli, green beans, chayote, and carrots. In Oaxaca City, participants also mentioned eating vegetables, *guia* (tender stems from zucchinis and chayotes), *comidas caldosas* (brothy meals), chicken soup, and pasta soup during their pregnancies.

Health issues and changes in their appetite varied for women and affected the amount and types of food consumed during this period. While more than a quarter of the participants mentioned they would continue to eat the same types of food when they were pregnant as they would before pregnancy, others did mention changing their habits. There was a contrast between the amount of food eaten during pregnancy, as several participants in Oaxaca and Puerto Escondido mentioned being very hungry during their pregnancy, while some participants in Puerto Escondido mentioned having trouble eating or keeping food down due to nausea. Some participants from Oaxaca City mentioned not consuming meat during their pregnancy due to nausea. Many participants in both cities mentioned that the food they ate was based on their pregnancy cravings, while several participants in Oaxaca City and Puerto Escondido mentioned eating food exclusively to have a healthy baby. Many of the participants from both Oaxaca City and Puerto Escondido mentioned eating what they refer to as “junk food” before getting pregnant, changing their eating habits during pregnancy, and continuing to eat healthy now that they have children. Only a few participants mentioned not following recommendations from physicians or family members on what to eat.

*“About three months or so*,* no*,* I hardly ate because he (the baby) made me very nauseous. Everything I ate*,* I would throw it up*,* I would throw it up and so on*,* so what*,* I did try*,* I tried to eat a little bit of fruit or drink some atole* (traditional hot beverage made from ground corn dough), *something like that so that it would more or less stay in my stomach*,* because even so I was vomiting” (Participant #12*,* age 31*,* Puerto Escondido)*.

Employment obligations and financial difficulties negatively impacted the nutrition practices of these women during pregnancy. Some of the participants in both locations mentioned that they worked during their first pregnancy, but because of work commitments, such as office work, housekeeping, and working at food stands and markets, they were unable to eat during their work hours. Five of them mentioned eating during their pregnancy what they considered to be quickly prepared and consumed, such as sandwiches and smoothies. Nearly a quarter of participants from both locations mentioned having financial difficulties during their pregnancy that directly impacted their means to buy food. One of these participants lived in the Oaxacan countryside at the time of her pregnancy and ate what was available, while the ones that lived in the city mentioned eating what was affordable at the time.

*“Well*,* (when pregnant) I would skip meals a bit*,* since I worked*,* I didn’t have time to eat sometimes.” (Participant #24*,* age 28*,* Puerto Escondido)*.

*”Well right now*,* right now I eat chepiles (a leafy vegetable) or sometimes when there is*,* well*,* like chicken*,* when there isn’t*,* well*,* some eggs*,* beans…(Why I didn’t eat that during pregnancy) Because sometimes I could not afford it.” (Participant #19*,* age 41*,* Puerto Escondido)*.

Related to nutritional knowledge, some participants have gained information from their own educational experience. For example, two participants from Puerto Escondido obtained it when they were in high school or college, with one of them having experienced leading nutritional workshops at her previous job. Many participants from both Oaxaca and Puerto Escondido mentioned that some of the nutritional knowledge comes from what their children are currently learning in school or what they themselves learned as students. A number of participants from Puerto Escondido mentioned using the internet to learn more about nutrition during pregnancy, such as by looking for new recipes.

When asked about nutritional supplement consumption, all participants mentioned they started consuming supplements, mostly folic acid and iron, after learning they were pregnant, with a number of participants learning about their pregnancy as late as the third to fifth month of gestation. All participants consumed nutritional supplements, in the form of iron, folic acid, or multivitamins, at least for one month. When the supplements were consumed, all participants mentioned they followed instructions and consumed them daily. Many participants, primarily from Puerto Escondido, consumed *Materna*, a multivitamin with folic acid, iron, calcium, and zinc, for pregnant and breastfeeding women available for a cost at pharmacies and not provided by public health care centers, or switched to *Materna* halfway through the pregnancy. Most participants who took *Materna* would take it until the end of their pregnancy. Excluding participants who consumed *Materna*, only a few participants consumed both iron and folic acid for their entire pregnancy, as nearly a quarter of the group would stop one or another after a few months. The main reasons for stopping nutritional supplement consumption during pregnancy were side effects (mainly vomit and constipation) or chronic health conditions (such as hemorrhoids), physician’s recommendations, and personal decisions.

*“I followed very precisely all my supplements*,* including pregnancy vitamins.” (Participant #20*,* age 34*,* Puerto Escondido)*.

Only a few participants mentioned that geographical distance or economic means were barriers to obtaining supplements. Some participants from both locations continued to consume some form of nutritional supplement, most commonly iron or vitamins, after giving birth, but one of the participants from Puerto Escondido reported stopping consumption, even when recommended by physicians, due to economic difficulties in acquiring the supplements.

*“Yes*,* because you have to come here because where we live there were no pharmacies*,* and we had to come here to buy it… And if there is no car*,* you have to wait 2 hours.” (Participant #6*,* age 24*,* Oaxaca City)*.

#### Theme 2. Female family members as primary source of nutritional knowledge and food support

For the majority of participants in both locations, the main source of nutritional knowledge and practices during pregnancy came from their older family members, particularly their mothers, grandmothers, and mothers-in-law. Examples of guidance provided by elder female family members included drinking *atole* (a traditional Mexican hot beverage made from ground corn dough), recommendations on how to deal with cravings, and avoiding pork, spicy foods, and certain seafood.

*“Well*,* older adults always tell you to “eat this because it is very good"” (Participant #2*,* age 31*,* Oaxaca City)*.

*”sometimes (my mom) would tell me " don’t eat that because it will make you sick” and sometimes I would be tempted but (I) said “no"… Because my baby comes first*,* my craving doesn’t matter” (Participant #15*,* age 30*,* Puerto Escondido)*.

Regardless of the participant’s age or the number of pregnancies they have had, mothers and grandmothers were also a support for many participants in food acquisition, as they cooked or brought food for participants during their pregnancy. Elder female family members also provided tips and recipes on what to cook with the ingredients available. Other examples of family support during pregnancy include reminding participants about taking nutritional supplements.

*“Well*,* my grandmother was the one who lived with us*,* since (other family member) worked*,* my grandmother stayed with me. She was the one who would get food and then if we got to eat sometimes*,* we would cook together” (Participant #3*,* age 29*,* Oaxaca City)*.

*“(our grandmother was) very attentive because she said (to prepare) an enchilada pobre (translated as enchilada for the poor)*,* that is not expensive but is delicious… she taught us that food that you have always liked*,* that she doesn’t have much*,* she only has garlic*,* tomato*,* onion and a little bit of cinnamon*,* and a guajillo chili*,* nothing more” (Participant #2*,* age 31*,* Oaxaca City)*.

#### Theme 3. Support from husbands and other members of women’s social network

The role and support from other members of the social network of these women during pregnancy were briefly described during the interviews. For example, participants’ partners or husbands were particularly mentioned regarding food acquisition. Food consumed by participants during pregnancy was mostly acquired by themselves or their partner. Most of the participants mentioned that while they were the ones who decided what to eat or buy, their partner was the one who would physically go grocery shopping or get takeout food.

*“My husband is the one who has worked*,* he is the one who brought us the groceries.” (Participant #1*,* age 38*,* Oaxaca City)*.

Some participants mentioned that their partners or former partners disliked the idea of them visiting health centers during the pregnancy, discouraging participants from attending a health facility to receive prenatal care and nutritional advice.

Participants’ fathers were mentioned a few times by supporting them financially, to pay for private health care services during pregnancy, or by providing food to the participants. Only three participants, one from Puerto Escondido and two from Oaxaca City, mentioned consuming the food they or their fathers grew, as most participants mentioned acquiring food at local markets, local butcher shops, and local produce stores.

Participants also mentioned obtaining referrals or recommendations on medical services to receive or request during pregnancy from friends and neighbors. This was more frequently related to the suggestion of consuming *Materna*, a brand of nutritional supplement, as a number of participants used or switched to this brand after the recommendation from family members or friends. Family members and friends who were physicians or former midwives were also sources of information for some participants in relation to nutritional guidance and knowledge.

#### Theme 4. Medical guidance for nutrition during pregnancy

Participants discussed extensively the nutritional guidance they received from health care providers during their pregnancy. Participants who received prenatal care from private physicians were the ones who were told more information about nutrition by their provider through their one-on-one prenatal care appointments. The most common pieces of information received were examples of foods to eat, such as vegetables, fruits, and soups, and foods to avoid during pregnancy, such as greasy food, pork, seafood, and spicy food. Some participants from Puerto Escondido mentioned that they received nutritional education through talks at the health center. Another participant from Puerto Escondido mentioned learning about nutrition through medical brigades when she lived in a small town in the state of Oaxaca.

*“(the doctor told me) about eating vegetables*,* eating a lot of vegetables*,* a lot of fruit. Gelatin*,* which is for bone calcification. A lot of legumes*,* um*,* drinking a lot of juices*,* a lot of liquids*,* so that both me and my baby*,* um*,* would get stronger. (Participant #1*,* age 38*,* Oaxaca City)*

Participants in both cities mentioned that the knowledge taught by health professionals was around the *el plato del bien comer* (“the eatwell plate”) (see Supporting material 3) [72], a food guide used by the Mexican government for nutritional guidance. Many participants in Oaxaca City mentioned knowing about *el plato del bien comer* through visual information such as educational flyers at health centers, while some participants from Puerto Escondido commented on how health professionals, specifically physicians and nurses, used *el plato del bien* comer to teach them about nutrition. Nearly a quarter of the participants also mentioned receiving materials about diets and foods to consume or not consume during their prenatal appointments without explanation about the diet or nutrition itself.

*“(the private doctor) taught me about the “plato del bien comer”. But it’s up to me*,* then. Yes*,* I knew that I had to eat well.” (Participant #24*,* age 28*,* Puerto Escondido)*.

Several participants from Puerto Escondido previously attended private nutritionists, when not pregnant, to lose weight and had gained nutritional knowledge from those appointments. While one participant from Puerto Escondido visited a dietitian during her pregnancy, due to her presenting gestational diabetes, while another participant from Oaxaca City received a nutritional orientation from a private endocrinologist during her pregnancy due to a thyroid condition.

*“I have thyroid (disease) and my son could have been born with some malformation or mental disability. So*,* they gave me a diet because I could not eat certain foods*,* coffee*,* I could not eat gluten.” (Participant #3*,* age 29*,* Oaxaca City)*.

#### Theme 5. Quality and gaps in the broader health care system and social services

The last theme identified corresponds to gaps in prenatal health services that directly or indirectly affected the nutritional knowledge and practices of women during pregnancy, which were common among participants. These include delayed care, difficulties obtaining nutritional supplements, and experience with different forms of social services. Delayed care in the public health care system was common, particularly in Oaxaca City, as many participants mentioned they would have to wait for the physician, who at times would not show up. Some participants mentioned that to avoid this, they would instead go to a pharmacy or a private physician to receive services, where they did not receive nutrition education.

Several participants had to move from receiving services from public to private health institutions during one of their pregnancies due to health conditions developed during the prenatal period. Some participants from Oaxaca City who started their prenatal care at a public health care setting switched during their pregnancy to receiving prenatal services from private physicians and facilities due to pregnancy complications. One participant in Oaxaca City who was in her second trimester with her second child at the time of the interview had recently started her prenatal care in a hospital that was hours away from her, as the staff from her local health center had been on leave and the participant had not received prenatal care for the first half of her pregnancy.

*“Well*,* at times*,* the doctor doesn’t come or*,* no*,* or sometimes the doctor comes late. They make us wait a long time.” (Participant #1*,* age 38*,* Oaxaca City)*.

Several participants who received prenatal care services in public health centers would have to buy nutritional supplements due to stock-outs at the health center. Some participants from both cities mentioned having to buy iron or folic acid at least once during their pregnancy due to stock-outs at the health center. Particularly in Oaxaca City, most women who had more than one pregnancy received their nutritional supplements without cost at a health center for their older child. For the participants in Oaxaca City who had a recent pregnancy, most had to buy their supplements, as these were no longer provided or were stocked out by the health center. In Puerto Escondido, nutritional supplement supply was almost always available for women who received prenatal care in health centers, for recent pregnancies and earlier pregnancies.

*“Just (have to buy) the pill (supplement)*,* the consultation is free and the pill when there is no (at the) clinic*,* they gave us a prescription to buy it.” (Participant #5*,* age 21*,* Oaxaca City)*.

Related to health insurance, almost none of the participants had health insurance at the time of the interview. Four participants mentioned they used to have *Seguro Popular*, Mexico’s social health protection system for people without access to private or work-based health insurance that was eliminated in 2020 [27]. When asked about their experience with *Prospera*, Mexico’s former Conditional Cash Transfer (CCT) program, some participants mentioned having received support from this program; however, they either received it after giving birth or when they were minors living with their parents. One participant from Oaxaca City who had received *Prospera* when she was already a mother mentioned that the monetary support was not used for nutritional purposes and was rather used to pay for their children’s school supplies. While other former *Prospera* participants remember receiving education classes, they did not specifically remember the health and nutrition education provided by this CCT program. A few participants from Oaxaca City mentioned the difficulties of being able to join *Prospera* due to the paperwork required and the involvement of political parties in the decision on who could join this CCT program. One participant who unsuccessfully tried to enroll in this CCT in the past suggested the need for *Prospera* to come back, as a lot of low-income people need this type of support.

*“Well*,* well*,* for the Prospera program to come back again.Yes*,* so that*,* well*,* more than anything*,* there are people who are low-income. Well*,* right now as now*,* a birth is expensive.” (Participant #7*,* age 27*,* Oaxaca City)*.

In relation to other social services used by participants, very few had experience with them. The ones who had received some form of social service received it in monetary form and were unsure of the name of the social program they were enrolled in or called it a scholarship. A few participants from Puerto Escondido described receiving economic support in the past as working mothers.

## Discussion

The objective of this qualitative study was to explore the nutritional practices of Oaxacan women during pregnancy, the nutritional education and knowledge obtained during this period, and their acquisition and use of prenatal supplements. We were able to put into context, using the Ecological Systems Theory and the Intersectionality Framework, how the different social positions of these women during pregnancy and the different relationships at the meso- and macro-levels influence their nutritional knowledge and practices. The present study demonstrates of the lived nutritional realities of women during pregnancy, shaped by their decisions and experiences, as well as those around them. Our findings show that women in Oaxaca City and Puerto Escondido primarily get basic nutritional knowledge during their pregnancy from family members and healthcare providers. Our results suggest that during pregnancy, women in Oaxaca City and Puerto Escondido have strong social support from their mothers, grandmothers, mothers-in-law, and partners. Nutritional practices and knowledge varied among women based on their working status, education, living location (including geographical location), and health issues during pregnancy, while for some, their prenatal care access and services varied based on their marital status and living location. This study also shed light on the use of nutritional supplements during pregnancy, as well as the different ways these are obtained by women in Oaxaca, depending on the prenatal care location, recommendations from family and healthcare providers, as well as preference and experience taking a specific brand.

The lived experiences of women residing in Oaxaca and Puerto Escondido revealed that their nutritional practices during the prenatal period varied based on the intersectionality of their multiple social positions during their pregnancy, such as work status, access to health care services and health insurance, education, and living location, to name a few. For example, several of the participants who were employed while pregnant mentioned skipping meals during their prenatal period because of work. This is consistent with prior research completed in Southeastern Mexico that suggests that skipping meals is associated with lower socioeconomic status and lower levels of educational attainment, as well as an intergenerational cycle of poverty [73]. Our participants also mentioned having financial difficulties during their pregnancy to access food. Close to 45% of women in Mexico live in poverty [74], while specifically in the state of Oaxaca, almost 33% of women lack access to nutritious and quality food [75]. However, specific information about economic vulnerability among pregnant women in Mexico, and particularly in the state of Oaxaca, is unknown. Several of the participants also mentioned geographical distance as an issue in obtaining nutritional supplements and accessing health care services during the prenatal period. The distance to travel was also found to be a main barrier, primarily for women, to use health services in Central Mexico [76]. The literature shows that maternal outcomes are influenced and interconnected by vulnerabilities, such as limited access to economic and healthcare resources [77]. While our results show that Oaxacan women are resilient and find ways to eat during pregnancy, such as growing their own food, finding ways to cook with the ingredients they have, and acquiring food from their family members, particularly their mothers, more research is needed to further understand how different vulnerabilities affect their pregnancy and the nutritional gaps these women have because of financial difficulties.

Themes one, two, and three, in particular, informed what types of foods were eaten by Oaxacan women during their pregnancy and the reasons behind these practices. As described for theme two, pork, spicy food, and certain seafoods were some of the main foods that family members, particularly mothers and grandmothers, told participants not to consume during their pregnancy. Pork, fats, and coffee have also been mentioned in the literature to be considered by Mexican women as harmful foods that should not be consumed during pregnancy [78–80]. *Atole* was a beverage recommended to participants by family members and health care professionals during the prenatal period. The limited literature on nutritional habits in pregnant women in Mexico has found that beans, fruit, vegetables, bread, *sopa de pasta* (a broth-based pasta soup), and *atole* are commonly consumed during the prenatal period in the central states of Zacatecas and Jalisco [78, 80]. Most of these foods were frequently mentioned by participants in Oaxaca City and Puerto Escondido as what they commonly consumed during their pregnancy. While the research in Jalisco focused specifically on teenagers, a population that faces high health risks during pregnancy [81], these findings, along with ones from the Zacatecas-based study, were similar to ours in terms of some women changing what they eat during pregnancy in order to have a healthy baby. Similar to our findings, a study among Mexican-born women living in the Northeastern United States (U.S.) also found that mothers who experienced their pregnancy in Mexico or the U.S. also transitioned their nutritional practices to foods of greater nutritional value. While the findings suggest that nutritional practices during pregnancy might be similar across the country and among Mexican women living in the U.S., future studies should be completed among the pregnant population in other states to better understand the dietary practices of women from different geographical regions during their prenatal period [82]. For example, while a recent study among men and women in Mexico suggests that people from Northern Mexico eat more fish, meat, and eggs and less maize and fruits than people from Southern and Central Mexico, this research excluded pregnant women [83]. Understanding the nutritional practices of women during pregnancy and whether these are maintained or change by geographical region could help local, state, and national programs tailor programs that follow the nutritional practices of each population.

Our findings highlight the importance of female family members, particularly mothers and grandmothers, in the participants’ pregnancies, related to guidance on nutritional practices as well as acquiring and preparing food. To our knowledge, this is the first study completed in the state of Oaxaca that studies the sources of nutritional knowledge during pregnancy, including knowledge and support from family members. Other studies in Mexico have also studied the support of mothers and grandmothers in maternal nutrition, but more particularly related to breastfeeding. Still, most of these results are similar to what we found in Oaxaca. For example, a mother-infant dyad study in the state of Yucatan found that receiving advice from grandmothers during the mothers’ pregnancy was positively associated with child nutritional status, and mothers without that type of support reached for emotional and informational support from other female relatives [84]. Two studies, one in the central state of Morelos and another one in three rural unnamed communities in Mexico, found that grandmothers and mothers-in-law are the leaders in the family in terms of health advice and promotion [85, 86]. A study in Indigenous communities in Central Mexico found that grandmothers provide nutritional knowledge gained from previous generations to their daughters and other women, regardless if they are part of their family or not [87]. The same study also found a difference in terms of breastfeeding and infant nutrition knowledge passed from grandmothers and health providers, which confuses mothers on what advice to use [87]. In our study, there were no contradictions between the nutritional information provided by mothers and grandmothers compared to healthcare providers. However, the objective of this research was not to compare nutritional knowledge between family members and healthcare professionals for pregnant women.

Related to the use of nutritional supplements during pregnancy, while all participants took some form of nutritional supplement for at least one month during their pregnancy, it was common for participants to stop consuming these after a few months. We believe this is the first qualitative study that explores nutritional supplement consumption during pregnancy in the state of Oaxaca. There are several quantitative studies that have been completed among Mexican pregnant women on either nutritional supplements or their iron levels. For example, low iron consumption among pregnant women has been found in Mexico City [88, 89]. These quantitative nutritional studies on pregnant women have been mostly completed in northern and central Mexico, while Oaxaca is located in southern Mexico, and the traditions among northern, central, and southern Mexico vary greatly [17, 78, 88, 89]. A study found that most pregnant women know about the nutritional requirements in iron and fiber needed during gestation; however, only half know about the protein and calcium required during this period [90]. While in our study we did not ask specifically about knowledge and consumption of different vitamins and minerals, as all of our participants consumed nutritional supplements for at least one month, our results suggest that women in Oaxaca are aware of the higher nutrient requirements during the prenatal period. In our study, many participants from both Oaxaca City and Puerto Escondido did not comply with nutritional supplement consumption for their entire pregnancy, and many, particularly in Oaxaca City, struggled to get nutritional supplements from local health centers. Also, some participants mentioned not starting their prenatal care and nutritional supplement consumption until the third to fifth month of pregnancy. These results match recent quantitative data that found that in the state of Oaxaca, none of the health units comply with adequate vitamin supplementation for pregnant adolescents, while close to 20% of women in Oaxaca do not start their prenatal care during the first trimester of their pregnancy [91, 92]. 

From our results, women, particularly in Oaxaca City, had gaps in their health services during the prenatal period that directly or indirectly affected their nutrition knowledge and practices and the acquisition and use of nutritional supplements. The literature has found that Mexican women living in rural areas, Indigenous women, those without health insurance, and women with lower socio-economic levels have worse coverage of the antenatal and delivery continuum of care, and there are greater gaps in health services during the prenatal and postnatal periods [25]. A recent study found that 20% of health units in Oaxaca comply with adequate follow-up with patients during pregnancy, still higher than other states such as Veracruz and Yucatan [91]. Most of our participants in this study did not have health insurance, and all were from lower socio-economic levels, confirming, at least in the capital city, that there are health disparities in the coverage of antenatal care for women with these characteristics.

### Strengths and limitations

To our understanding, this is the first qualitative study to explore the nutritional knowledge and practices of Oaxacan women during pregnancy. A strength of this study was the use of different theoretical frameworks (Ecological Systems Theory and the Intersectionality Framework) and an inductive analytical methodology (Grounded Theory) to inform the interview guide, data coding and analysis, allowing for data collection and result analysis to be guided by different lenses grounded in participant experience. While there are some studies related to nutrition in Mexico using the Ecological Systems Theory, particularly related to breastfeeding [93, 94], to our knowledge, this is the first research in Mexico that uses the Intersectionality Framework as well as an adaptation of both frameworks (the Ecological Systems Theory and the Intersectionality Framework) in nutrition during pregnancy. While there is a vast literature related to food insecurity in Mexico [95–97], including specific to the state of Oaxaca [98], most research related to food accessibility and consumption excludes pregnant women from their measurement. Our research provides specific information based on the experience of women in their pregnancy and yields evidence to understand the nutritional inequalities they suffer during the prenatal period based on their position in society, such as their working status, living location, and education.

One potential limitation of this study was the small number of participants interviewed in the city of Oaxaca due to recruitment challenges. However, data saturation was reached. Also, while interviews were completed in rooms to secure participant privacy, some of the women who participated had different distractions, such as having to take care of the children, which could have potentially led to under-reporting of nutrition knowledge and practices during pregnancy. Recall bias is another potential limitation to this study, as participants were asked about their nutritional practices during all of their pregnancies, and those whose first pregnancies happened over a longer time interval could potentially not accurately remember. Also, women who had given birth more than once could potentially combine their experiences, leading to inaccurate recollections of nutrition practices during a specific pregnancy. Finally, while the study was conducted in two cities of the state of Oaxaca, recruitment in the city of Puerto Escondido was exclusive to women present at one health center, and results from this study might not be representative of other urban areas or in rural areas in the state of Oaxaca or in other regions in Mexico.

## Conclusions

A healthy diet during pregnancy is necessary for the short- and long-term health outcomes of the mother and the child. It is essential to understand the nutrition knowledge and practices women follow during the prenatal period for policies and programs to address potential barriers and enhancers to achieving the daily nutritional requirements during pregnancy. The results of this study are consistent with the Intersectionality Framework and the Ecological Systems Theory, as the nutritional knowledge and practices of Oaxacan women during pregnancy are impacted by the multiple social positions and characteristics women have and the different factors at the individual, group, and structural level, respectively. While health care professionals provide nutritional information for women during pregnancy, our results highlight the strong support from family members, particularly mothers and grandmothers, in providing nutritional knowledge and helping obtain or prepare food during this period. While women in Oaxaca consume nutritional supplements during pregnancy, only some consume them for their entire pregnancy. Future research should further examine the prevalence of micronutrient deficiency among Oaxacan women during pregnancy to better understand the effect of limited nutritional supplement intake during the prenatal period. Learning from the perspective of health care professionals in the public and private sectors about how nutrition education is delivered and the misconnections between provider and patient related nutrition during this important time is a critical aspect to further pursue. Finally, while women rely on the support of family and health care providers, their own life experiences and decisions shape their nutritional knowledge and practices during the prenatal period. Our study provides a foundation for the knowledge and practices of Oaxacan women during pregnancy; however, additional research is required to further understand the potential gender and social gaps to address the nutritional inequities these women present during pregnancy.

## Electronic supplementary material

Below is the link to the electronic supplementary material.

Supplementary Material 1

## Data Availability

Participants’ transcripts are not available to share as they contain confidential information.

## Abbreviations

C-section: Cesarean section
CCT: Conditional Cash Transfer
ELCSA: Latin American and Caribbean Scale of Food Security
U.S.: United States
WHO: World Health Organization
