# Supplementary material for: Nutritional knowledge and practices of low-income women during pregnancy: a qualitative study in two Oaxacan cities
Source: J Health Popul Nutr. 2025 Feb 7;44:33. doi: 10.1186/s41043-025-00776-8 (PMC11806553; doi:10.1186/s41043-025-00776-8)
Supplement: Supplementary file 1 — Supplementary Material 1 [file 41043_2025_776_MOESM1_ESM.docx]

**Supporting materials**

1. **Semi-structured interview guide**

| **Related to food consumption and acquisition:**   1. What would you say were the foods you ate the most during the last pregnancy? What are the reasons for consuming these foods? What were you told by your doctor/person providing care to eat during your pregnancy? 2. What would you say are the foods you eat most frequently? What are the reasons for consuming these foods? 3. Did you eat differently during your pregnancies? If so, how do you eat differently? 4. (Only for women who have been pregnant more than once) If has changed, what are the reasons for that? 5. Do you eat differently when you are pregnant versus when you are not pregnant? If so, how do you eat differently? 6. How did you get the food that you ate when you were pregnant? How do you get the food (when not pregnant)? |
| --- |
| **Related to nutrition education:**   1. What type of nutrition education did you receive when you were pregnant and from whom? 2. What type of nutrition education have you received (when not pregnant) and from whom? 3. Where would you say your nutrition knowledge comes from? |
| **Related to nutritional supplements:**   1. Did you consume nutritional supplements during your pregnancy? 2. If no to Q11, what are the reasons for not consuming nutritional supplements during your pregnancy? 3. If yes to Q11, what type of supplements did you took? How did you obtain these supplements? How frequently would you say you consume these supplements? For how long did you consume these supplements? For the ones who stopped consuming nutritional supplements, could you provide me the reason why you stopped taking the supplements? |
| **Related to *Prospera* and other social services:**   1. Did you previously receive services from *Prospera*? 2. If yes to Q14, what type of nutrition education did you receive from *Prospera*? 3. If yes to Q14, did you receive services from *Prospera* when you were pregnant? 4. If yes to Q16, what type of nutrition education did you receive from *Prospera* when you were pregnant? 5. Did you receive nutritional supplements from *Prospera* when you were pregnant? 6. If yes to Q18, what type of supplements did you receive? How often did you receive these? How frequently did you consume these supplements? For how long did you consume these supplements? For the ones who stopped consuming nutritional supplements, why did you stop consuming the supplements? 7. How was your nutrition different when you were pregnant and receiving *Prospera* compared to your last pregnancy? (for women who received *Prospera* while pregnant and were pregnant at least once when they were not receiving *Prospera*) 8. Did you receive any other form of social services when you were pregnant? If yes, what services were those? What type of nutritional education did you receive from these? For how long? |

1. **Additional quotes**

| **Theme** | **Quote** |
| --- | --- |
| 1. Life experiences, sociodemographic, and health characteristics that influence nutritional practices and knowledge during pregnancy | *"I had problems because I felt insecure and when I went to work, I sometimes skipped meals, I didn't give me time to eat." (Participant #3, age 29, Oaxaca City)*  *"Well, the truth is, well, I did suffer for a while for money" (Participant #7, age 27, Oaxaca City)*  *"Well, since school. From school they teach us how to eat, right? They take us, they give us classes, maybe in the books it comes. And the talks they normally give you." (Participant #27, Puerto Escondido)* |
| 1. Female family members as a primary source of knowledge and food support | *"From older family members, well, the elders. They are the ones who tell you "no, you can't eat this"" (Participant #25, age 41, Puerto Escondido)*  *"And at times, (the pregnancy) asked me for acidic stuff (food), but at times they (grandmother and mother) would not allow me, because they said that when the baby is born it will hurt you a lot. And then, the baby would grow with, how do you say? With stomach inflammation" (Participant #13, age 30, Puerto Escondido)*  *"Well, from time to time my mother brings me food, mole (Mexican dish with a traditional sauce of the same name), broths. But, well, most of the time, I do them." (Participant #26, age 21, Puerto Escondido* |
| 1. Support from husbands and other members of women’s social network | *“I hardly saw the doctor there…because my children's father was a little more closed. So, no, he almost didn't like me going to the doctor.” (Participant #14, age 34, Puerto Escondido)*  *“And here we don't know, if it is high risk (pregnancy) we don't want to be responsible for what happens." So this one, well then my dad was there, says “no daughter, he says, you better go to an individual, he won't do what your baby costs you, but make sure it turns out well.” (Participant #1, age 38, Oaxaca City)*  *“(I use Materna nutritional supplement) because my husband's aunt was taking that. And during both of her pregnancies she was taking that…That's very good, I can still use it while breastfeeding.” (Participant #22, age 22, Puerto Escondido)* |
| 1. Medical guidance for nutrition during pregnancy | *"(Nutritionist taught me that) I have to eat in portions, I have to eat snacks, that I should not eat so much fat, no sugar, not with so much salt. And, well, also, eat five times a day, have snacks, reduce carbohydrates, and all that" (Participant #27, age 34, Puerto Escondido)*  *"Here (At the health center) the nurses just (taught) about the "plato del bien comer" and all of that"*  *(Participant #27, age 34, Puerto Escondido)*  *"(the doctor told me) to eat zucchinis, well, green beans, chayotes, all of the vegetables" (Participant #4, age 20, Oaxaca City)* |
| 1. Quality and gaps the broader health care system and social services | *“I almost always resort more to Similares (a pharmacy), because at the (health) center it takes them all day to see…It's difficult, to waste time” (Participant #2, 31, Oaxaxa City)* |

1. **Plato del bien comer (the eatwell plate)**

**
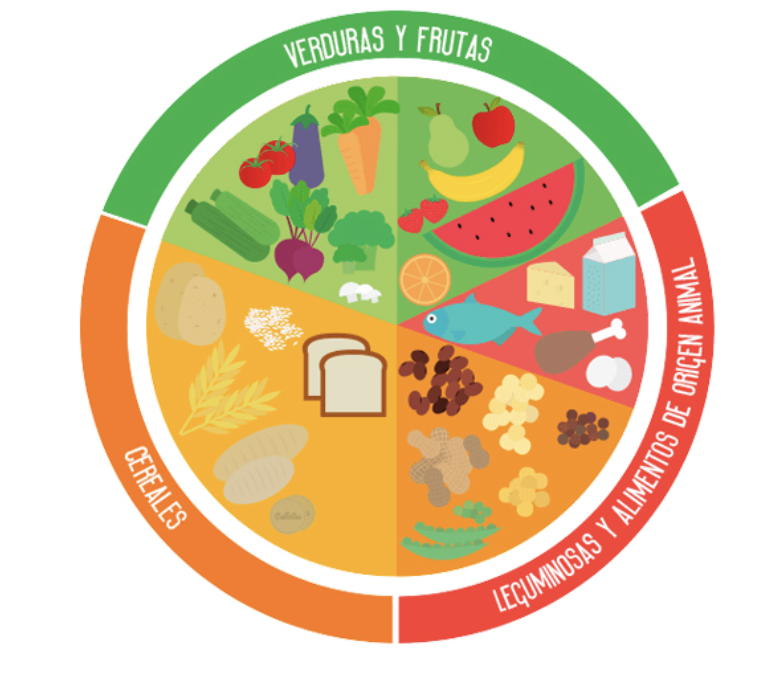
**
